# Undergraduate nursing students’ preparedness for transition to internship: A scoping review protocol

**DOI:** 10.1371/journal.pone.0349467

**Published:** 2026-08-21

**Authors:** Jennifer Buckley, Sarah Costello, Marcella Horrigan Kelly, Lisa Sweeney, Emma Tynan

**Affiliations:** Department of Nursing and Healthcare, Technological University of the Shannon, Athlone, County Westmeath, Ireland; School of Nursing Sao Joao de Deus, Evora University, PORTUGAL

## Abstract

**Background:**

The transition from undergraduate nursing student to internship is viewed as a critical stage in professional development. During this transitional period, students are expected to consolidate clinical competence, integrate theoretical knowledge with their practice, and develop confidence and readiness for increasing responsibility in healthcare provision. Internship or internship-like experiences are embedded within undergraduate nursing curricula internationally, and it is recognised that this transition is often associated with uncertainty, anxiety, and reduced confidence. Recent developments in Irish nursing education, particularly the Registered Nurse Education Programme Standards published by the Nursing and Midwifery Board of Ireland [NMBI], have strengthened the emphasis on readiness for practice and by inference internship preparation. A review and synthesis of contemporary evidence is therefore needed to map what is known about undergraduate nursing students’ preparedness for transition to internship in preparation for future practice as a qualified nurse.

**Objective:**

This scoping review aims to identify and map the breadth of evidence addressing undergraduate nursing students’ preparedness for transition from supernumerary clinical placements to internship, including identification of the factors that either facilitate or hinder student preparedness to support this transition.

**Methods:**

This protocol follows Joanna Briggs Institute [JBI] scoping review methodology and uses the Population, Concept, and Context [PCC] framework to guide eligibility criteria, search strategy, screening, extraction, and final synthesis of the evidence. Reporting for this protocol will follow the Preferred Reporting Items for Systematic Reviews and Meta-Analyses protocols [PRISMA-P]. The completed scoping review will adhere to the guidance of the Preferred Reporting Items for Systematic Reviews and Meta-Analyses Extension for Scoping Reviews [PRISMA-ScR] to ensure transparency and rigour in final reporting. The protocol has been registered on the Open Science Framework available at https://osf.io/9zdgy/. A pilot search will be conducted in CINAHL and ERIC to refine search terms. A comprehensive search will then be undertaken in CINAHL, MEDLINE via PubMed, Scopus, and ERIC, including grey literature sources from the Health Service Executive, Department of Health, Higher Education Authority, ProQuest Dissertations and Theses Global, EMBASE Conference Abstracts, and Conference Proceedings Citation Index. Screening and data charting will be conducted independently in research team pairs using Covidence. Extracted data will be analysed and synthesised using a descriptive and thematic approach with the overall aim of synthesis being to map the nature and characteristics of the evidence.

**Dissemination:**

Findings will be disseminated through peer-reviewed publication and conference presentation.

## Introduction

Internationally, undergraduate nursing education programmes commonly include internship or internship-like clinical experiences that are intended to facilitate the transition from student status to professional nurse status. These experiences are designed to support the consolidation of clinical competence, integration of theory and practice, and development of confidence before registration as a nurse [1–6].

In Ireland, the undergraduate nursing programme includes a final-year clinical internship that is regarded as a key component to facilitate transition from supernumerary learner under direct supervision to intern working with indirect supervision [5]. Recent and forthcoming changes to Irish undergraduate nursing curricula further emphasise internship, reflecting a focus on community-based internship experiences, aligning with broader healthcare reform and regulatory requirements for practice readiness [6–8]. The Irish context echoes the international focus supporting progression from novice to advanced beginner. Canadian programmes include consolidated final-year placements or preceptorships, United States programmes commonly include capstone internships, and the United Kingdom and Australia include supervised practice experiences and transition-focused models [1–4,9]. Thus, from both the international and Irish context, the expectation is that nursing students assume increased responsibility in developing their clinical decision-making, competence, confidence, professional identity, and readiness to deliver safe patient centred care as part of a transitional phased process [10–12].

While this phased transition is viewed as crucial in terms of development for practice readiness, inherent challenges are recognised during this phase of professional development [13–15]. The literature has described this transitional period using concepts such as “reality shock” and “transition shock,” reflecting the emotional, intellectual, and professional demands experienced by learners [13,14]. Students may encounter uncertainty, anxiety, reduced confidence, and pressure linked to increased accountability and changing role expectations [15,16]. In the Irish context, contemporary developments in nurse education have increased the importance of understanding preparedness for internship. The Registered Nurse Education Programme Standards emphasise competency-based education and require that curricula demonstrate students’ readiness for practice at the point of registration [6]. This creates a strong rationale for examining the breadth and nature of available evidence relating to undergraduate nursing students’ preparedness for transition to internship in preparation for their future role as registered nurses.

This scoping review will include evidence published between 2016 and 2026 to capture literature that reflects contemporary undergraduate nursing education, current approaches to internship preparation, and recent regulatory expectations for practice readiness [2,5,6,8,17,18]. This timeframe was selected because the review aims to inform current and future curriculum development, clinical education strategies, transition-support interventions, and internship planning within undergraduate nursing programmes. Although seminal literature published before 2016 has made key contributions to understanding the theoretical concepts underpinning preparedness for transition to internship, including clinical competence, experiential learning, transition shock, and progression from student to registered nurse [10,13–16,19], these works will primarily inform the theoretical and contextual background of the review rather than the evidence mapped.

Undergraduate nursing education, professional regulation, and healthcare delivery have evolved substantially over the past decade, with increased emphasis on competency-based curricula, simulation and technology-enhanced learning, person-centred care, patient safety, supervision and preceptorship, professional accountability, and readiness for practice [2,5–9,16]. Consequently, restricting the review to evidence published from 2016 to 2026 is considered appropriate to ensure that the findings reflect contemporary educational, regulatory, and clinical contexts. It is acknowledged that this review’s timeframe has the potential to exclude earlier studies that remain relevant to preparedness for transition to internship. However, the timeframe was chosen to ensure that this review reflects contemporary undergraduate nursing education, current internship preparation, and recent regulatory developments. This limitation will be considered when interpreting the findings and acknowledged in the completed review [17,18].

For the purposes of this review, preparedness for transition to internship is understood as a complex, multidimensional, and developmental concept. As the literature describes preparedness using a range of related concepts and dimensions rather than a single consistent definition, an operational definition was developed by the review team specifically to guide study identification, eligibility assessment, data extraction, and evidence mapping [2,4,11–16]. This operational definition was informed by Benner’s Novice-to-Expert model, Steinaker and Bell’s experiential taxonomy, and contemporary literature relating to readiness for practice and transition to professional nursing [2,4,10–16,19]. Benner’s Novice-to-Expert model provides the theoretical basis for understanding the progressive development of clinical competence, clinical judgement, confidence, responsibility, and professional accountability through clinical experience, while Steinaker and Bell’s experiential taxonomy explains how these capabilities develop through progressive experiential learning [10,19]. Together with the contemporary literature and current regulatory expectations for undergraduate nursing education, these perspectives inform the progression of undergraduate nursing students during the latter stages of their programme as they move from advanced beginner supernumerary status under direct supervision towards advanced beginner internship under distant or indirect supervision [2,4–6,11–16,19]. Preparedness is therefore operationally defined in this review as “a dynamic and fluid transitional process through which the undergraduate nursing student is exposed to experiential learning to integrate and develop knowledge, skills, and professional judgement while moving from advanced beginner supernumerary status under direct supervision to advanced beginner internship under distant or indirect supervision.”

For this review, preparedness will be operationalised as a multidimensional concept encompassing clinical competence, confidence, self-efficacy, professional identity, clinical judgement, experiential learning, readiness for increasing responsibility and accountability, and the organisational and educational factors that may facilitate or hinder the transition to internship. These dimensions were selected because they are consistently reported across the literature and align with current regulatory expectations for safe, competent, and accountable nursing practice [2,4–6,11–16,19]. This operational definition will provide a structured approach for study identification, data extraction, and evidence mapping.

### Review questions

The primary review question is: What is known about nursing students’ preparedness for the transition from supernumerary clinical placements to internship?

The secondary review question is: What factors facilitate or hinder undergraduate nursing students’ preparedness for this transition?

### Objectives

The objectives of this scoping review are to explore the current evidence on undergraduate nursing students’ preparedness for transition to internship and identify factors that facilitate or hinder preparedness, examine interventions, programmes, and strategies designed to support transition to internship to inform future research and educational practice.

## Methods

### Review design

This study is a scoping review designed to identify and map the breadth of evidence addressing nursing students’ preparedness for internship. A scoping review methodology is viewed as appropriate for this research as it facilitates mapping of key concepts and types of evidence linked with the transitional phase of moving from directed supervision to indirect supervision as a student nurse [17,18,20,21]. The review will be conducted in accordance with the Joanna Briggs Institute methodology framework. The Population, Concept, and Context [PCC] framework was used to guide the development of the review questions, eligibility criteria and search strategy, and will be used to ensure clarity for all elements of this study’s review processes [17,18]. Reporting for this protocol will follow the Preferred Reporting Items for Systematic Reviews and Meta-Analyses protocols [PRISMA-P] [22] (see S6 Appendix). For the completed scoping review reporting will follow PRISMA-ScR guidance to ensure transparency and rigour [23] (see S6 Appendix).

### Protocol registration

The protocol has been registered on the Open Science Framework as follows: Horrigan Kelly M, Buckley J, Sweeney L, Tynan E, Costello S. Undergraduate Nursing Students’ Preparedness for Transition to Internship: A Scoping Review Protocol [24] available at https://osf.io/9zdgy/

### Review stages and timeframe

The protocol development and OSF registration has been completed in March 2026; an initial pilot search was conducted in April 2026; and post-pilot refinement of the research question, search terms, and eligibility criteria was completed in April 2026. A full search will be conducted from April to July 2026. Screening, review and selection will be conducted between July to September 2026. Data extraction and charting will be conducted from September to October 2026. Data analysis and synthesis using descriptive thematic analysis will be undertaken from November 2026 to January 2027. Write-up of the scoping review and findings from same will be completed from January to March 2027. Preparation for publication submission and dissemination will be undertaken from March to June 2027 with the planned end date for this review being June 2027.

### Eligibility criteria

The eligibility criteria for this review were developed by the research team using the PCC framework [17,18]. The population is undergraduate nursing students in third-level education. Studies focused on postgraduate nursing students or qualified nurses will be excluded. The concept is preparedness for transition to internship and includes practice readiness, transition from supernumerary to intern status, including concepts such as competence development, experiential learning, and role transition. Studies focused on postgraduate internship and postgraduate preparation will be excluded. The context is undergraduate nursing education, focusing on internship preparation, transition to internship, and internship-related clinical learning. Studies focused on postgraduate nursing education programmes will be excluded.

The timeframe for included sources is between 2016 and 2026 with sources excluded before 2016 or after 2026. This timeframe was selected to capture evidence that reflects contemporary undergraduate nursing education, current approaches to internship preparation, and recent regulatory and clinical practice contexts relevant to practice readiness. Earlier seminal literature will inform the theoretical and contextual background of the review, while the evidence mapping will focus on contemporary literature most applicable to current undergraduate nursing education and internship preparation. Only studies published in English or translatable to English will be included.

Evidence sources will include peer-reviewed qualitative, quantitative, and mixed-methods research. Relevant grey literature will also be included to capture policy, regulatory, educational, and organisational evidence that may not be available through peer-reviewed publications. Eligible grey literature may include government and regulatory reports, professional guidance, national or institutional policy documents, strategic reports, curriculum materials, dissertations, theses, conference abstracts, and conference proceedings. All evidence sources will be screened using the same Population, Concept, and Context (PCC) eligibility criteria. Studies focused on postgraduate nursing students or qualified nurses, postgraduate nursing education, internship or clinical placement, studies not addressing preparedness for transition to internship, or studies not related to supernumerary roles, internship, or transition to practice will be excluded.

Consistent with Joanna Briggs Institute guidance, formal methodological quality or risk-of-bias appraisal will not be undertaken. Instead, grey literature will undergo a structured eligibility and credibility assessment to determine its suitability for inclusion in the review. Documents will be assessed to ensure that they are relevant to the review question, originate from an identifiable and appropriate author or organisation, provide sufficient information to support meaningful data extraction, and meet the review eligibility criteria and timeframe. Editorials, letters, commentaries, blogs, promotional material, duplicate records, and documents without identifiable authorship or sufficient information to determine their relevance will be excluded. Where multiple versions of the same document are identified, the most complete and relevant version will be retained. Further details of the inclusion and exclusion criteria are provided in S1 Appendix.

### Information sources and search strategy

The databases that will be searched for this scoping review include: CINAHL, ERIC, MEDLINE, and Scopus. These will be accessed through CINAHL via EBSCOhost, ERIC via EBSCOhost, MEDLINE via PubMed, and Scopus via the Elsevier Scopus interfaces. Grey literature will be searched through the Health Service Executive, Department of Health, Higher Education Authority, ProQuest Dissertations and Theses Global, EMBASE Conference Abstracts, and Conference Proceedings Citation Index. The search strategy is guided by the PCC framework and will be refined through an initial pilot search in two databases, these being CINAHL and ERIC. The focus of the pilot search is to identify relevant keywords, indexing terms, and concepts across the literature, and to refine if required the final search strategy prior to the full search being conducted. The core search concepts are population terms relating to undergraduate nursing students; concept terms relating to preparedness, readiness, transition, experiential learning, novice to expert, advanced beginner, and clinical competence; and context terms relating to internship, nursing internship, clinical internship, supernumerary, preceptorship, capstone, and transition programme. These will be combined with Boolean operators. Search strings will be adapted to the subject headings of each respective database and limited to publications from 2016 to 2026 available in English-language or translatable to English. The search strategy reflecting the PCC framework, mapping of search terms and database application is provided in S1 Appendix. S1 Appendix provides an example of a draft search strategy developed for MEDLINE [via PubMed] to demonstrate the approach that will be employed across all databases. This draft search strategy incorporates both Medical Subject Headings [MeSH] and free-text terms. Boolean operators [AND, OR] to combine search terms, and truncation [*] that will be applied where appropriate to capture variations in terminology. This strategy will be piloted and refined in both CINAHL and ERIC to ensure sensitivity and relevance. The finalised search strategy will then be adapted for use across all the databases, including CINAHL, MEDLINE, Scopus, and ERIC, with appropriate modifications to subject headings and syntax.

### Screening and selection of sources

Covidence data management system will be used to support screening, review, and selection of sources. Identified records from the searches will be imported into Covidence. Zotero reference management system will be used to export data in RIS format for import into Covidence. Duplicates will be removed using the automated system in Covidence before screening. Two stages will be involved in the screening of data, firstly title and abstract, secondly full-text screening. Screening stages will be conducted independently in pairs by members of the review team adhering to the inclusion and exclusion criterion for this research (see S1 Appendix). Screening will be blinded in Covidence restricting reviewers’ access to one another’s decisions. Discrepancies will be resolved through discussion and consensus. Where agreement cannot be reached, the broader review team will act as third reviewer. To ensure consistency, reviewers will adhere to the inclusion and exclusion criterion outlined in the reviewer instructions (see S2 Appendix). All sources meeting the eligibility criteria will proceed to the data extraction phase. The study selection process will be reported using the PRISMA flow diagram [23] (see S5 Appendix).

### Data extraction and charting

For data extraction the charting forms developed by the review team for both empirical evidence and grey literature will be used (see S2 Appendix [empirical studies] and S3 Appendix [grey literature]). Data extraction will be conducted independently by two reviewers working in pairs. Following extraction, paired reviewers will compare charted data side by side. Discrepancies will be resolved through discussion, reference to the original study, and consultation of the predefined eligibility criterion. Where necessary, the broader team will act as third reviewer to resolve disagreements with agreed changes documented.

### Critical appraisal of included sources

A formal assessment of risk of bias or methodological quality of the included studies will not be undertaken as the aim of this review is to map the breadth and nature of the available evidence. This approach is reflective with guidance advocated by the Joanna Briggs Institute for scoping reviews.

### Data management and sharing

On completion of the review, outputs such as the study characteristics tables, screening outcomes, and PRISMA documentation will be shared on the Open Science Framework repository in keeping with transparency and reproducibility principles [24] (see S2–S5 Appendices).

### Outcomes and variables of interest

It is anticipated that this scoping review has potential to identify a diverse body of evidence addressing preparedness for transition to internship for nursing students. The primary outcome of interest is undergraduate nursing students’ preparedness for transition to internship. Variables of interest include factors that facilitate or hinder preparedness, such as educational strategies, simulation, curriculum design, mentorship, preceptorship, supervision models, clinical learning environments, organisational supports, and individual student characteristics. This review will examine interventions and structured programmes designed to support transition, including capstone experiences, internship preparation initiatives, and clinical support mechanisms. Outcomes will be mapped using descriptive thematic analysis to reveal how preparedness is defined, described, and revealed across the literature.

### Synthesis

Data synthesis will reflect a descriptive thematic analytical approach [17,20,21,25]. As the purpose of this scoping review is to map the breadth and nature of the available evidence rather than undertake an in-depth interpretive analysis, descriptive thematic analysis will be used to identify and organise recurring patterns across the literature. Qualitative and narrative findings will be organised using a descriptive thematic analytical process informed by Braun and Clarke’s [25] six-phase framework that encompasses: familiarisation with the extracted data, systematic coding, the identification, review and refinement of descriptive themes, and the reporting of findings. This analytical framework will be used as a structured approach to familiarisation with the data, coding, development and refinement of descriptive categories, and presentation of recurring themes, rather than to generate an interpretive theory. Qualitative data management software will not be used. Instead, coding and organisation of extracted findings will be undertaken using the predefined data-charting forms within Covidence and the review team’s data extraction process.

Initially, study characteristics, populations, contexts, and interventions will be presented in both tabular format and narrative summarisation. Two reviewers will independently undertake open coding of extracted findings. Similar codes will be grouped into descriptive categories before being synthesised into recurring descriptive themes representing dimensions of preparedness, barriers, facilitators and educational interventions. Theme development will be iterative, with regular discussion among reviewers and refinement through consensus. Any disagreements will be resolved through discussion and, where necessary, consultation with a third reviewer. Quantitative findings will be summarised descriptively and integrated narratively with qualitative findings. Findings will be coded and grouped into recurring themes related to preparedness, facilitating and hindering factors, and interventions that support transition to internship. Emerging codes, thematic groupings, and interpretations will be discussed and refined through team-based consensus. Divergent interpretations will be addressed by revisiting the extracted data and review objectives until agreement is reached. Reporting of the review will follow PRISMA-ScR guidelines (see S6Appendix).

### Ethics and dissemination

Ethical approval is not required because this review will synthesise data from publicly available peer-reviewed and grey literature sources. No personal or sensitive data will be collected. The review will be conducted in accordance with recognised principles of research integrity, transparency, and academic rigour and is aligned with institutional expectations for responsible research conduct [26]. Findings will be disseminated through submission to a peer-reviewed journal and presentation at relevant conferences.

### Software

Covidence will be used for screening, selection, and data extraction. Zotero will be used for reference management.

## Supporting information

S1 Appendix Eligibility criterion, Mapping Search Terms Data Base Application and Sample Search using MEDLINE [via PubMed]. (DOCX)

S2 Appendix Reviewer instructions for screening and selection. (DOCX)

S3 Appendix Data charting form for empirical studies. (DOCX)

S4 Appendix Data charting form for grey literature. (DOCX)

S5 Appendix PRISMA 2020 flow diagram. (DOCX)

S6 Appendix PRISMA-P checklist & PRISMA-ScR checklist. (DOCX)
