# Supplementary material for: Undergraduate nursing students’ preparedness for transition to internship: A scoping review protocol
Source: PLoS One. 2026 Aug 21;21(8):e0349467. doi: 10.1371/journal.pone.0349467 (PMC13496437; doi:10.1371/journal.pone.0349467)
Supplement: Supplementary file 1 [file pone.0349467.s001.docx]

Appendix S1

This appendix provides an overview of the eligibility criteria for this scoping review, including the inclusion and exclusion criteria, mapped search terms, and database-specific application notes. It also includes an example search strategy for MEDLINE via PubMed to illustrate the Population–Concept–Context framework operationalisation.

Inclusion and exclusion criterion, Mapping of Search Terms and Data Base Application

The eligibility criteria and search strategy for this scoping review **will be developed** using the Population–Concept–Context [PCC] framework. This appendix presents the inclusion and exclusion criteria alongside the corresponding search terms and application to the databases that **will be used.**

| **Domain [PCC]** | **Inclusion Criteria** | **Exclusion Criteria** | **Mapped Search Terms** | **Database Application / Notes** |
| --- | --- | --- | --- | --- |
| **Timeframe** | Studies published between 2016 and 2026 will be included | Studies published before 2016 or after 2026 will be excluded | Not applicable [limiters] | Date limits re timeframe will be applied across all databases |
| **Language** | Studies published in English or translatable to English will be included | Studies not in English and not translatable will be excluded | Not applicable [filters] | English language filters will be applied |
| **Population** | Undergraduate nursing students in third-level education will be included | Postgraduate nursing students and qualified nurses will be excluded | "undergraduate nursing student*" OR "nursing student*" OR "BSc nursing" OR "baccalaureate nursing" OR "degree nursing student*" OR "third level nursing student*" OR "Level 8 QQI nursing student*" | Subject headings [e.g., MeSH, CINAHL Headings] will be used when available |
| **Context** | Undergraduate nursing programmes, internship preparation, clinical education, and transition to internship will be included | Postgraduate education and post graduate nursing programmes will be excluded | "internship" OR "clinical internship" OR "nursing internship"  OR "capstone" OR "transition programme" OR "nursing apprenticeship" OR "clinical placement" | Search terms will be tailored to reflect the rules of language of each data base |
| **Concept** | Preparedness for transition to internship, experiential learning, transition from supernumerary to intern roles, and competence development will be included | Postgraduate internship, postgraduate clinical placement will be excluded | "preparedness" OR "readiness" OR "transition" OR "transition to practice" OR "transition to internship" OR "clinical competence" OR "experiential learning" OR "role transition" OR "novice to expert" OR "advanced beginner" OR "supernumerary" OR "intern" OR "supervised trainee nurse" | Concept terms will be combined using Boolean operators [AND/OR] |
| **Study Types [Peer-reviewed]** | Quantitative, qualitative, mixed methods, case studies, case reports, randomised controlled trials, literature reviews, reviews of reviews, and qualitative evidence syntheses will be included | Editorials, opinion pieces, commentaries, letters will be excluded | Search limiters will not be applied | Eligibility criteria for this scoping review protocol will be applied during the screening stage |
| **Grey Literature** | Policies, reports, strategic documents, guidance documents, protocols, and clinical updates will be included | Editorials, opinion pieces, commentaries, letters re grey literature will be excluded | "policy" OR "report" OR "guideline" OR "strategy" OR "protocol" OR "clinical update" | Searches will be conducted across HSE, Department of Health, HEA, ProQuest, and conference databases |
| **Search Strategy Structure** | The PCC framework will be applied to structure all searches | Not applicable | [Population] AND [Concept] AND [Context] | Boolean operators will be used across all databases listed below |
| **CINAHL [EBSCOhost]** | Searches will include undergraduate nursing students, preparedness/transition and internship-related context terms within the timeframe and outlined and reflecting English language limits as outlined | Records not meeting the inclusion criterion will be excluded | [MH "Students, Nursing, Baccalaureate"] AND [MH "Clinical Competence"] AND [MH "Internship Programs"] including keyword equivalents | Limits that will be applied include the eligibility criterions of Language: English and the timeframe of 2016–2026 |
| **MEDLINE [PubMed]** | Searches will include undergraduate nursing students, preparedness/transition and internship-related context terms within the timeframe and outlined and reflecting English language limits as outlined | Records not meeting the inclusion criterion will be excluded | ["Students, Nursing"[MeSH]] AND ["Clinical Competence"[MeSH]] AND ["Internship "[MeSH]] including keyword terms | Filters that will be applied include the eligibility criterions of Language: English and the timeframe of 2016–2026 |
| **Scopus** | Searches will include undergraduate nursing students, preparedness/transition and internship-related context terms within the timeframe and outlined and reflecting English language limits as outlined | Records not meeting the inclusion criterion will be excluded | Search terms will be applied to the title, abstract, and keyword fields [TITLE-ABS-KEY], and combined using Boolean operators Articles will be included if they include the specified search terms reflecting the PCC framework within the title, abstract, or keywords. | Limits that will be applied is linked with the inclusion criterion of English and timeframe of 2016–2026 |
| **ERIC [EBSCOhost]** | Searches will include undergraduate nursing students, preparedness/transition and internship-related context terms within the timeframe and outlined and reflecting English language limits as outlined | Records not meeting the inclusion criterion will be excluded | ["nursing students"] AND ["preparedness" OR "transition"] AND ["internship" OR "capstone"] | Limits that will be applied is linked with the inclusion criterion of English and timeframe of 2016–2026 |

Example Search Strategy [MEDLINE via PubMed]

This appendix presents a draft search strategy developed for MEDLINE (via PubMed) to demonstrate the approach that will be applied across all databases. The search strategy is constructed using the Population–Concept–Context (PCC) framework. The population component will include terms related to undergraduate nursing students. The concept component will include terms relating to preparedness, readiness, transition, and competence development. The context component will include terms relating to internship, clinical placement, and transition-focused educational experiences.

The following search strategy will be applied:

**POPULATION**

("Students, Nursing"[MeSH] OR "nursing student*" OR "student nurse*" OR "undergraduate nursing student*" OR "baccalaureate nursing” OR "degree nursing student*" OR "third level nursing student*" OR "Level 8 QQI nursing student*")

AND

**CONCEPT**  
("Clinical Competence"[MeSH] OR preparedness OR readiness OR transition* OR "transition to practice" OR "transition to internship" OR "role transition” OR "practice readiness" OR "clinical preparedness" OR "experiential learning" " OR "clinical competence" OR "novice to expert" OR "advanced beginner" OR "supernumerary" OR "intern" OR "supervised trainee nurse”)

AND

**CONTEXT** (["Internship" OR "clinical internship" OR "nursing internship" OR capstone OR "clinical placement*" OR "practice placement*" OR supernumerary OR "transition programme" OR "nursing apprenticeship")

The search will be limited to studies published between 2016 and 2026, and to articles published in English or translatable to English. No study design limits will be applied at the search stage. This strategy will be piloted and refined in both MEDLINE and CINAHL to ensure sensitivity and relevance The finalised search strategy will then be adapted for use across all included databases, MEDLINE, CINAHL, Scopus, and ERIC, with appropriate modifications to subject headings and syntax.
