# Supplementary material for: Undergraduate nursing students’ preparedness for transition to internship: A scoping review protocol
Source: PLoS One. 2026 Aug 21;21(8):e0349467. doi: 10.1371/journal.pone.0349467 (PMC13496437; doi:10.1371/journal.pone.0349467)
Supplement: Supplementary file 2 [file pone.0349467.s002.docx]

**Appendix S2. Screeners Guidance/Instruction for Reviewers**

The following is guidance to the research review team regarding screening of the studies for this scoping review. This guidance is to ensure we are consistent, transparent, and rigorous in our approach to study selection. Also to ensure we are screening in accordance with the Population–Concept–Context [PCC] framework and our defined eligibility criterion. Screening is conducted using Covidence data management software. Table one below provides the inclusion and exclusion criterion applicable to our scoping review

**ELIGIBILITY CRITERION- INCLUSION AND EXCLUSION**

| **INCLUSION CRITERIA** | **EXCLUSION CRITERIA** |
| --- | --- |
| Timeframe 2016-2026 | Pre 2016, Post 2026 |
| English or Translatable to English | Non-English or Not Translatable to English |
| **Participants-** | |
| Nursing Student  Undergraduate Nursing Student | Post Graduate Nursing Students |
| **Context-** | |
| Undergraduate Nursing Degree in Third Level Education, Internship Education and Preparation | Postgraduate Nursing Degree in Third Level Education |
| **Concept-** | |
| Preparedness for Transition to Internship  Supernumerary  Intern | Post graduate intern  Post graduate internship  Post graduate clinical placement  Post graduate preparation |
| **All peer reviewed evidence**  Quantitative research studies  Qualitative research studies  Mixed methods studies  Case studies  Case reports  RCT  Literature Reviews  Review of Reviews  Qualitative Evidence Synthesis [QES] | Non peer reviewed articles |
| Strategic focused materials  Published Strategies  Reports  Policies  Guidance Documents  Strategies,  Clinical updates  Case reports,  Protocols | Editorials  Opinion pieces  Comment pieces  Letters  Reviews not including reported data |

**General Advice**

Apply eligibility criteria consistently across all records. 
When uncertain at title/abstract stage, include for full-text review. 
Maintain independent and blinded screening. 
Do not discuss decisions until both reviewers have completed screening.

**Title and Abstract Screening**

Adhere strictly to the eligibility criterion included  
 
***Exclude studies that:*** 
In accordance with the exclusion criterion

**Full-Text Screening**

Assess full texts against all eligibility criteria: 

**Conflict Resolution**

Conflict/ discrepancies where possible should be resolved through discussion between paired reviewers. If resolution cannot be reached consensus should be sought from the broader research team members

**Documentation**

Accurate records are imperative to ensure PRISMA-ScR reporting is fulfilled. All exclusions at full text stage screening should be recorded with rationale for same in Covidence to ensure the PRISMA chart is an accurate representation of the screening process.
