# Supplementary material for: Undergraduate nursing students’ preparedness for transition to internship: A scoping review protocol
Source: PLoS One. 2026 Aug 21;21(8):e0349467. doi: 10.1371/journal.pone.0349467 (PMC13496437; doi:10.1371/journal.pone.0349467)
Supplement: Supplementary file 3 [file pone.0349467.s003.docx]

Appendix S3. Data charting form for empirical evidence

| **Data Charting [Empirical Evidence]** | | | | | | | | |
| --- | --- | --- | --- | --- | --- | --- | --- | --- |
| **DATA EXTRACTION DETAILS** | | | | | | | | |
| **Author**  **Year of publication** | **Country/**  **Region** | **Title of paper/report**    **Journal title** | **Aims/Purpose of study** | **Study type/ Design** | **Study population and Sample Size** | **Results** | **Outcome/s of Interest** | **Comments re applicability to this scoping review study** |
|  |  |  |  |  |  |  |  |  |
|  |  |  |  |  |  |  |  |  |
|  |  |  |  |  |  |  |  |  |
|  |  |  |  |  |  |  |  |  |
