# Supplementary material for: Undergraduate nursing students’ preparedness for transition to internship: A scoping review protocol
Source: PLoS One. 2026 Aug 21;21(8):e0349467. doi: 10.1371/journal.pone.0349467 (PMC13496437; doi:10.1371/journal.pone.0349467)
Supplement: Supplementary file 4 [file pone.0349467.s004.docx]

Appendix S4. Data charting form for grey literature

| **Data Charting [Grey Literature]** | | | | | | | | | |
| --- | --- | --- | --- | --- | --- | --- | --- | --- | --- |
| **DATA EXTRACTION DETAILS** | | | | | | | | | |
| **Author**  **Year of publication** | **Country/**  **Region** | **Publication Title** | **Publication Type** | **Publication Location**  **Link to full text** | **Aims/Purpose of publication** | **Population** | **Concept** | **Context** | **Comments/ Relevant Findings/ Recommendations or Conclusions relevant to this scoping review** |
|  |  |  |  |  |  |  |  |  |  |
|  |  |  |  |  |  |  |  |  |  |
|  |  |  |  |  |  |  |  |  |  |
|  |  |  |  |  |  |  |  |  |  |
|  |  |  |  |  |  |  |  |  |  |
|  |  |  |  |  |  |  |  |  |  |
|  |  |  |  |  |  |  |  |  |  |
