# Supplementary material for: Undergraduate nursing students’ preparedness for transition to internship: A scoping review protocol
Source: PLoS One. 2026 Aug 21;21(8):e0349467. doi: 10.1371/journal.pone.0349467 (PMC13496437; doi:10.1371/journal.pone.0349467)
Supplement: Supplementary file 6 [file pone.0349467.s006.docx]

**Appendix S6**

This appendix includes both the PRISMA-P checklist for reporting items in systematic review and meta-analysis protocols and the PRISMA-ScR checklist, which extends PRISMA reporting guidance for scoping reviews.

**PRISMA-P Checklist**

**Preferred reporting items for systematic review and meta-analysis protocols [PRISMA-P] 2015 statement.**

| **Section/topic** |  | **Checklist item** | **Information reported** | | **Line number[s]** | |
| --- | --- | --- | --- | --- | --- | --- |
|  |  |  | **Yes** | **No** |  |  |
| **ADMINISTRATIVE INFORMATION** | | | | | |  |
| **Title** | | | | | |  |
| Identification | 1a | Identify the report as a protocol of a systematic review | Y |  | P1, Title | |
| Update | 1b | If the protocol is for an update of a previous systematic review, identify as such |  | N | Not applicable | |
| Registration | 2 | If registered, provide the name of the registry [e.g., PROSPERO] and registration number in the Abstract  Horrigan Kelly, M., Dr., Buckley, J., Sweeney, L., Tynan, E., & Costello, S. [2026, March 25]. Undergraduate Nursing Students’ Preparedness for Transition to Internship: A Scoping Review Protocol. [https://doi.org/10.17605/OSF.IO/9ZDGY](https://scanner.topsec.com/?d=2120&r=show&u=https%3A%2F%2Fdoi.org%2F10.17605%2FOSF.IO%2F9ZDGY&t=6f0273ce14ff1670a09176fd9aa8c286a06d04ad) | Y |  | P5, Protocol Registration | |
| **Authors** | | | | | |  |
| Contact | 3a | Provide name, institutional affiliation, and e-mail address of all protocol authors; provide physical mailing address of corresponding author | Y |  | P1, Title Page | |
| Contributions | 3b | Describe contributions of protocol authors and identify the guarantor of the review |  | N | Not reported | |
| Amendments | 4 | If the protocol represents an amendment of a previously completed or published protocol, identify as such and list changes; otherwise, state plan for documenting important protocol amendments | Y |  | Page 8 Data Management and Sharing  All future amendments will be documented on OSF [https://doi.org/10.17605/OSF.IO/9ZDGY](https://scanner.topsec.com/?d=2120&r=show&u=https%3A%2F%2Fdoi.org%2F10.17605%2FOSF.IO%2F9ZDGY&t=6f0273ce14ff1670a09176fd9aa8c286a06d04ad) | |
| **Support** | | | | | |  |
| Sources | 5a | Indicate sources of financial or other support for the review | Y |  | P9, Funding  No funding has been received for this scoping review. | |
| Sponsor | 5b | Provide name for the review funder and/or sponsor |  | N | Not applicable | |
| Role of sponsor/funder | 5c | Describe roles of funder[s], sponsor[s], and/or institution[s], if any, in developing the protocol |  | N | Not applicable | |
| **INTRODUCTION** | | | | | |  |
| Rationale | 6 | Describe the rationale for the review in the context of what is already known | Y |  | P3 Introduction | |
| Objectives | 7 | Provide an explicit statement of the question[s] the review will address with reference to participants, interventions, comparators, and outcomes [PICO] | Y |  | P4-5 Review questions / Objectives  and Design reflecting PCC framework | |
| **METHODS** | | | | | |  |
| Eligibility criteria | 8 | Specify the study characteristics [e.g., PICO, study design, setting, time frame] and report characteristics [e.g., years considered, language, publication status] to be used as criteria for eligibility for the review | Y |  | P5-6 Review stages and timeframe, eligibility criteria and also S1 appendix | |
| Information sources | 9 | Describe all intended information sources [e.g., electronic databases, contact with study authors, trial registers, or other grey literature sources] with planned dates of coverage | Y |  | P6-7 Information sources and search strategy and also SI, S1A appendix | |
| Search strategy | 10 | Present draft of search strategy to be used for at least one electronic database, including planned limits, such that it could be repeated | Y |  | P6 Information sources and search strategy and S1A appendix | |
| **STUDY RECORDS** | | | | | |  |
| Data management | 11a | Describe the mechanism[s] that will be used to manage records and data throughout the review | Y |  | P7 Data management include Covidence, Zotero | |
| Selection process | 11b | State the process that will be used for selecting studies [e.g., two independent reviewers] through each phase of the review [i.e., screening, eligibility, and inclusion in meta-analysis] | Y |  | P7 Screening and selection of sources and also S1, S2 and s5 appendix | |
| Data collection process | 11c | Describe planned method of extracting data from reports [e.g., piloting forms, done independently, in duplicate], any processes for obtaining and confirming data from investigators | Y |  | P8 Data extraction and charting and also S2 and S3 appendix | |
| Data items | 12 | List and define all variables for which data will be sought [e.g., PICO items, funding sources], any pre-planned data assumptions and simplifications | Y |  | P8 Outcomes and variables and also S2–S4 Appendix | |
| Outcomes and prioritization | 13 | List and define all outcomes for which data will be sought, including prioritization of main and additional outcomes, with rationale | Y |  | P8 Outcomes and variables | |
| Risk of bias in individual studies | 14 | Describe anticipated methods for assessing risk of bias of individual studies, including whether this will be done at the outcome or study level, or both; state how this information will be used in data synthesis | Y |  | P8 Critical appraisal of included sources | |
| **DATA** | | | | | |  |
| Synthesis | 15a | Describe criteria under which study data will be quantitatively synthesized |  | N | Not applicable | |
|  | 15b | If data are appropriate for quantitative synthesis, describe planned summary measures, methods of handling data, and methods of combining data from studies, including any planned exploration of consistency [e.g., *I* ^2^, Kendall’s tau] |  | N | Not applicable | |
|  | 15c | Describe any proposed additional analyses [e.g., sensitivity or subgroup analyses, meta-regression] |  | N | Not applicable | |
|  | 15d | If quantitative synthesis is not appropriate, describe the type of summary planned | Y |  | P9 Synthesis -descriptive thematic analysis and synthesis | |
| Meta-bias[es] | 16 | Specify any planned assessment of meta-bias[es] [e.g., publication bias across studies, selective reporting within studies] |  | N |  | |
| Confidence in cumulative evidence | 17 | Describe how the strength of the body of evidence will be assessed [e.g., GRADE] |  | N | Not applicable | |

PRISMA-ScR checklist

**Preferred Reporting Items for Systematic reviews and Meta-Analyses extension for Scoping Reviews [PRISMA-ScR] Checklist**

| **SECTION** | **ITEM** | **PRISMA-ScR CHECKLIST ITEM** | **REPORTED ON PAGE #** |
| --- | --- | --- | --- |
| **TITLE** | | | |
| Title | 1 | Identify the report as a scoping review. | 1 |
| **ABSTRACT** | | | |
| Structured summary | 2 | Provide a structured summary that includes [as applicable]: background, objectives, eligibility criteria, sources of evidence, charting methods, results, and conclusions that relate to the review questions and objectives. | 1 |
| **INTRODUCTION** | | | |
| Rationale | 3 | Describe the rationale for the review in the context of what is already known. Explain why the review questions/objectives lend themselves to a scoping review approach. | 2-4 |
| Objectives | 4 | Provide an explicit statement of the questions and objectives being addressed with reference to their key elements [e.g., population or participants, concepts, and context] or other relevant key elements used to conceptualize the review questions and/or objectives. | 3 |
| **METHODS** | | | |
| Protocol and registration | 5 | Indicate whether a review protocol exists; state if and where it can be accessed [e.g., a Web address]; and if available, provide registration information, including the registration number. | 3 https://osf.io/9zdgy/ |
| Eligibility criteria | 6 | Specify characteristics of the sources of evidence used as eligibility criteria [e.g., years considered, language, and publication status], and provide a rationale. | 4; S1 Appendix |
| Information sources | 7 | Describe all information sources in the search [e.g., databases with dates of coverage and contact with authors to identify additional sources], as well as the date the most recent search was executed. | 4-7; S1 Appendix |
| Search | 8 | Present the full electronic search strategy for at least 1 database, including any limits used, such that it could be repeated. | 4-7; S1 Appendix |
| Selection of sources of evidence | 9 | State the process for selecting sources of evidence [i.e., screening and eligibility] included in the scoping review. | 5; S2-5 Appendix |
| Data charting process | 10 | Describe the methods of charting data from the included sources of evidence [e.g., calibrated forms or forms that have been tested by the team before their use, and whether data charting was done independently or in duplicate] and any processes for obtaining and confirming data from investigators. | 5; S2 & S3 Appendices |
| Data items | 11 | List and define all variables for which data were sought and any assumptions and simplifications made. | 5-6 |
| Critical appraisal of individual sources of evidences | 12 | If done, provide a rationale for conducting a critical appraisal of included sources of evidence; describe the methods used and how this information was used in any data synthesis [if appropriate]. | 6 |
| Synthesis of results | 13 | Describe the methods of handling and summarizing the data that were charted. | 6;  S6 Appendix |
| **RESULTS** | | | |
| Selection of sources of evidence | 14 | Give numbers of sources of evidence screened, assessed for eligibility, and included in the review, with reasons for exclusions at each stage, ideally using a flow diagram. | Planned; S5 Appendix |
| Characteristics of sources of evidence | 15 | For each source of evidence, present characteristics for which data were charted and provide the citations. | Planned; Page 6 |
| Critical appraisal within sources of evidence | 16 | If done, present data on critical appraisal of included sources of evidence [see item 12]. | Not applicable |
| Results of individual sources of evidence | 17 | For each included source of evidence, present the relevant data that were charted that relate to the review questions and objectives. | Planned [post-review] |
| Synthesis of results | 18 | Summarize and/or present the charting results as they relate to the review questions and objectives. | Planned [post-review] |
| **DISCUSSION** | | | |
| Summary of evidence | 19 | Summarize the main results [including an overview of concepts, themes, and types of evidence available], link to the review questions and objectives, and consider the relevance to key groups. | Planned [post-review] |
| Limitations | 20 | Discuss the limitations of the scoping review process. | Planned [post-review] |
| Conclusions | 21 | Provide a general interpretation of the results with respect to the review questions and objectives, as well as potential implications and/or next steps. | Planned [post-review] |
| **FUNDING** | | | |
| Funding | 22 | Describe sources of funding for the included sources of evidence, as well as sources of funding for the scoping review. Describe the role of the funders of the scoping review. | Page 6 |
