# Supplementary material for: Undergraduate nursing students’ preparedness for transition to internship: A scoping review protocol
Source: PLoS One. 2026 Aug 21;21(8):e0349467. doi: 10.1371/journal.pone.0349467 (PMC13496437; doi:10.1371/journal.pone.0349467)
Supplement: Supplementary file 7 [file pone.0349467.s005.docx]

Appendix S5. PRISMA 2020 flow diagram
